# NT-proBNP in cardiopulmonary resuscitated patients treated with mild therapeutic hypothermia is not independently associated with mortality: a retrospective observational study

**DOI:** 10.1186/s12871-015-0023-y

**Published:** 2015-04-09

**Authors:** Bob Smit, Angelique ME Spoelstra-de Man, Armand RJ Girbes, Monique C de Waard

**Affiliations:** Department of Intensive Care, Institute for Cardiovascular Research (ICaR-VU), VU University Medical Center, Amsterdam, Netherlands

**Keywords:** Cardiac arrest, Mild therapeutic hypothermia, Natriuretic peptides, NT-proBNP

## Abstract

**Background:**

In spite of the introduction of mild therapeutic hypothermia (MTH), mortality rates remain high in patients with return of spontaneous circulation (ROSC) after cardiac arrest (CA). To date, no accurate and independent biomarker to predict survival in these patients exists. B-type natriuretic peptide (BNP) was found to provide both prognostic and diagnostic value in various cardiovascular diseases, including survival to hospital discharge in patients with ROSC. However, the biologically inactive counterpart of BNP, NT-proBNP, was found to be a more stable and accurate analyte. The current retrospective observational study investigates the value of NT-proBNP to predict 28-day mortality in post-CA patients treated with MTH, as well as the dynamics of NT-proBNP during MTH.

**Methods:**

NT-proBNP levels were measured in post-CA patients cooled via cold intravenous saline infusion and water-circulating body wraps (Medi-Therm®, Gaymar). Plasma samples were obtained before cooling was started, at the start and end of the maintenance phase and at the end of rewarming.

**Results:**

250 patients, admitted between 2009 and 2013, had NT-proBNP levels measured on ICU admission and were included for the evaluation of NT-proBNP as a prognostic marker. In the 28 days following ICU admission, 114 patients died (46%). Non-survivors had significantly higher NT-proBNP (median 1448 ng/l, IQR 366–4623 vs median 567 ng/1, IQR 148–1899; *P* < 0.001) levels on ICU admission. Unadjusted odds ratios for 28-day mortality were 1.7 (95% CI 0.8-3.5), 1.6 (0.8-3.3) and 3.6 (1.7-7.5) for increasing quartiles of NT-proBNP as compared to the lowest quartile. Adjusted odds ratios were 1.1 (95% CI 0.5-2.5), 1.1 (0.5-2.5) and 1.6 (0.7-3.8), respectively. A cut-off value of 834 ng/l achieved a sensitivity of 58% and a specificity of 58% to predict 28-day mortality. Of 113 patients, NT-proBNP values of each MTH phase were available and grouped in decreased or increased levels in time. Both decreases and increases of NT-proBNP values were observed during the MTH phases, but presence of either was not associated with outcome.

**Conclusions:**

High NT-proBNP plasma concentrations on ICU admission are associated with high 28-day mortality in post-CA patients treated with MTH in a univariate analysis, but not in a multivariate analysis. Increases or decreases of NT-proBNP levels during MTH appear unrelated to 28 day mortality.

## Background

Mild therapeutic hypothermia (MTH) is now considered an established treatment to limit neurological injury and improve survival in patients who have been successfully resuscitated after cardiac arrest (CA) [1]. Despite the introduction of MTH, the mortality rate in successfully cardiopulmonary resuscitated patients after CA remains high. In Europe, survival rates are only 9-23% after out of hospital cardiac arrest [2]. Better prognostication is needed to identify high and low risk patients and subsequently intensify or reconsider treatment [3]. To date, no reliable predictive marker for outcome after CA exists. Natriuretic peptides, such as B-type natriuretic peptide (BNP) and N-terminal proBNP (NT-proBNP), are useful markers for diagnosis and prognosis of congestive heart failure and other cardiovascular diseases. In 2007 it was shown that BNP is of prognostic value in CA patients [4,5].

BNP and NT-proBNP are both end-stage products of the proBNP hormone, which is predominantly produced in the atrial and ventricular myocardium after transcription of the natriuretic peptide precursor B (NPPB) gene [6]. ProBNP is then enzymatically cleaved into BNP and NT-proBNP, which are both secreted into the circulation in an approximate 1:1 ratio. NT-proBNP is biologically inactive, while BNP exerts various effects such as the induction of vasodilation, increased diuresis and natriuresis.

Ventricular wall-stress [6], hypoxia [7-9] and ischemia-reperfusion induced inflammation [10,11], results in production of NT-proBNP and BNP. Although BNP is removed via receptor-binding and enzymatic cleavage, clearance of NT-proBNP is dependent on renal secretion [12]. Some studies show that cerebral ischemia also induces release of NT-proBNP and BNP [13-15]. Despite the influence of several CA-related events, such as hypoxic brain injury, ischemia-reperfusion injury and renal dysfunction after return of spontaneous circulation, on the expression of NT-proBNP, it potentially holds additional prognostic information in comparison to BNP.

In regard to pre-analytical and analytical conditions, NT-proBNP has several advantages over BNP. NT-proBNP is considerably more stable at room temperate and current laboratory assays are highly sensitive and specific [16]. Furthermore, all commercially available NT-proBNP assays utilize the same set of antibodies, which greatly simplifies intra-laboratory comparisons. Due to its renal clearance, NT-proBNP also has a much longer in-vivo half time than BNP (1–2 hours versus 20 minutes respectively), which reduces the influence of specific time-frame sampling, but, is therefore less sensitive for acute changes. Taken together, NT-proBNP may has the potential to exceed the prognostic value of BNP.

In the current retrospective observational study the prognostic value of NT-proBNP for survival of patients who were admitted to the ICU for treatment with MTH after CA, as well as the dynamics of NT-proBNP levels during hypothermia were investigated.

## Methods

### Study population

Patients with return of spontaneous circulation after all cause cardiac arrest, eligible for MTH treatment and admitted to the ICU of the VU University medical centre (Amsterdam, the Netherlands) between January 2009 and March 2013 were included. Exclusion criteria were cardiac arrest due to submersion or asphyxia, traumatic brain injury, and <18 years of age. Patients were treated according to a strict protocol of MTH which includes the use of cooled saline intravenous infusion and non-invasive surface cooling using body wraps (Medi-Therm, Gaymar, USA). After admission to the ICU, body wraps were placed around the patient’s body and cooling was started immediately. An external temperature control unit adjusts the temperature of the water circulating through the body wraps. An esophageal temperature probe was inserted for continuous temperature monitoring and connected to the temperature control unit for automated temperature feedback control. Blood samples were taken according to the phase of cooling (induction, maintenance and rewarming). The induction phase, till target temperature of 32.5°C was reached, was followed by the maintenance phase of 24 hours at a core temperature of 32.5°C. Thereafter the patients were gradually rewarmed to normothermic core temperature of 36.5°C at a rate of ≤ 0.5°C/hour. According to Dutch legislation, no approval of the research ethics committee is required for this standard treatment. The research ethics committee METc VUmc approved the anonymous use of the patient data for this retrospective study.

### Data retrieval

Data were obtained from a prospective registry database involving all patients at the ICU after CA treated with MTH. Demographics (age and gender), pre-existing comorbidities (diabetes mellitus (DM), congestive heart failure (CHF), cardiovascular disease history (CVD) and renal insufficiency), arrest conditions (cause of arrest, location, witnessed and ventricular fibrillation/tachycardia (VF/VT) as the initial rhythm), laboratory parameters (NT-proBNP, Troponin-T, Creatine Kinase Myoglobin (CK-MB), Creatinine, Urea, Lactate and pH) and APACHE II score were recorded in our patient data management system for critical care (Metavision, iMDsoft®, Israël). Survival at 28 days after ICU discharge was obtained from the patients hospital file.

### NT-proBNP measurements

**Figure 1:**
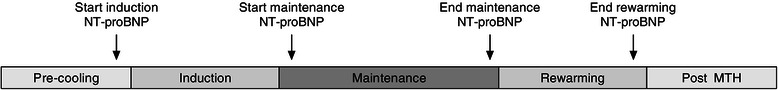
**NT-proBNP measurement time points.** NT-proBNP levels were measured at four different time points: 1) after admission to the ICU, prior to cooling 2) during the maintenance phase, after reaching a target temperature of 32.5°C, 3) at the end of the maintenance phase, before the initiation of rewarming 4) at the end of the rewarming phase.

Blood samples were collected from arterial lines, as part of standard care, on the following time points; 1) after admission to the ICU, prior to cooling 2) during the maintenance phase, after reaching a target temperature of 32.5°C 3) at the end of the maintenance phase, before the initiation of rewarming 4) at the end of the rewarming phase (Figure 1). NT-proBNP plasma levels were assessed on an ELECSYS 1010 bench top immunoassay analyser (Roche Diagnostics, Almere, Netherlands).

### Statistical analysis

Continuous data are presented as median and interquartile range (25^th^ to 75^th^ percentile). Unpaired data were compared using Mann–Whitney U-tests. A change in paired data was detected using Wilcoxon matched-pairs signed-rank test. Dichotomous data are presented as counts and percentages, comparisons were made using Fishers exact test.

After dividing patients into increasing NT-proBNP quartiles, binary logistic regression was performed to assess the prognostic value of NT-proBNP. To investigate the independent prognostic value of NT-proBNP, additional parameters were added to the regression model if there appeared to be a differential distribution between the survivors and non-survivors (defined as *P* < 0.20). Finally, a Receiver operating characteristic (ROC) curve was constructed to determine an optimal cut-off value.

Repeated measures obtained in the same patient during the different MTH phases were analysed by one-way repeated measures ANOVA and Bonferroni’s post-hoc correction to identify inter-phase NT-proBNP level changes.

*P-*values below 0.05 were considered as statistically significant. Calculations were performed using SPSS 20 (IBM Corporation, New York, USA). Graphs were made using GraphPad Prism 5.0 (GraphPad Software Inc, San Diego, USA).

## Results

### Patients

A total of 469 patients were admitted to the ICU for post-CA MTH treatment between January 2009 and August 2013. Of these patients, 150 met one or more of the exclusion criteria. From the remaining 319 patients, 250 had NT-proBNP levels measured in the period between ICU admission and induction of MTH and were included for analysis. Patient characteristics were divided into survivors and non-survivors (Table 1). During the 28 days following admission, 114 (46%) patients died. The median age of non-survivors was significantly higher compared to survivors (68 years; IQR 61–76 versus 61 years; IQR 53 – 70, *P =* 0.002). The percentage of witnessed arrests, initial rhythm VF/VT and cardiac cause of arrest in non-survivors was significantly lower than in survivors. Non-survivors had significantly lower initial body temperature, higher APACHE II score, shorter length of stay and lower GCS score compared to survivors. Survivors had lower NT-proBNP, creatinine, Urea, and lactate levels upon ICU admission compared to non-survivors (Table 2).

**Table 1:** **Patient characteristics**

	Survivors		Non-survivors	
	n = 136 (54%)		n = 114 (46%)	
**Demographics**				
Age (years)	61	(53–70)	68	(61–75)*
Female, n (%)	36	(26)	36	(32)
Body weight (kg)	80	(70–90)	80	(70–88)
Body Mass Index	25	(23–28)	25	(23–28)
**History of Illness**				
Diabetes, n (%)	11	(8)	21	(18)
CVD history, n (%)	59	(43)	58	(51)
Congestive heart failure, n (%)	19	(14)	22	(19)
Renal insufficiency, n (%)	10	(7)	14	(12)
**Cardiac arrest conditions**				
Out of hospital cardiac arrest, n (%)	121	(89)	95	(83)
Witnessed arrest, n (%)	106	(78)	75	(66)*
Initial rhythm VF/VT, n (%)	97	(71)	51	(45)*
Cardiac Cause, n (%)	112	(82)	76	(67)*
**In-hospital intervention/assessment**				
CAG, n (%)	73	(54)	56	(49)
PCI, n (%)	44	(32)	34	(30)
CABG, n (%)	5	(4)	1	(1)
APACHE II score	29	(24–34)	35	(29–39)*
ICU Length of stay (days)	7	(4–13)	4	(3–9)*
GCS on ICU discharge	14	(11–15)	3	(2–5)*
**Mild Therapeutic Hypothermia**				
Initial body temperature (°C)	34.9	(34.2–35.6)	34.5	(33.9–35.2)*
Cooling speed (°C/hour)	0.6	(0.3–0.8)	0.5	(0.2–0.8)
Rewarming speed (°C/hour)	0.3	(0.2–0.3)	0.3	(0.2–0.3)

* = *P* < 0.05 for inter-group comparisons (Mann Whitney U-test for continuous data, Fisher’s Exact test for dichotomous data). CVD, Cardiovascular disease; VF/VT, Ventricular Fibrillation/Tachycardia; CAG, Coronary Angiogram; APACHE, Acute Physiology and Chronic Health Evaluation; GCS, Glasgow Coma Score.

**Table 2:** **Laboratory parameters upon ICU admission**

	Survivors		Non-survivors	
	n = 136 (54%)		n = 114 (46%)	
NT-proBNP (ng/l)	567	(146–1813)	1448	(366–4623)*
Troponin-T (μg/l)	0.05	(0.02–0.17)	0.07	(0.03–0.41)
Troponin-T above 99^th^ percentile (>0.014 μg/l)	79	%	89	%
CK (U/l)	360	(157–910)	259	(130–820)
MB (μg/l)	7.8	(5.0–33.1)	10.1	(5.8–45)
Urea (mmol/l)	7.1	(5.9–9.4)	8.5	(6.6–11.2)*
Creatinine (μmol/l)	89	(75–107)	104	(82–128)*
Lactate (mmol/l)	4.8	(2.3–8.3)	7.4	(4.4–11.6)*
pH (lowest value)	7.3	(7.2–7.3)	7.3	(7.2–7.3)

* = *P* < 0.05 for inter-group comparisons (Mann Whitney U-test). NT-proBNP, N-terminal pro Brain Natriuretic Peptide; CK, Creatine Kinase; MB, Myoglobin.

### NT-proBNP and survival

**Figure 2:**
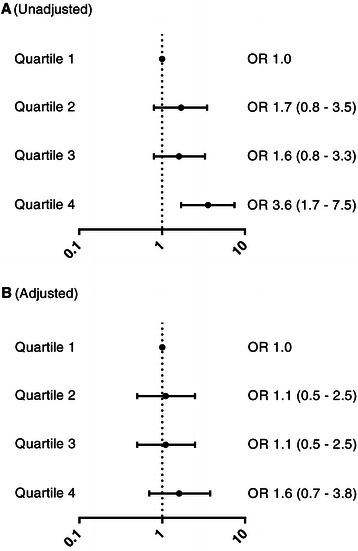
**Odds ratios for 28-day mortality.** Unadjusted **(A)** and adjusted odds ratios **(B)** for 28-day mortality in increasing quartiles. Adjusted odds ratios were calculated by correcting for age, diabetes mellitus, ventricular fibrillation/tachycardia as the initial rhythms and cardiac cause of arrest. Data are presented as OR (95% Confidence Interval). Abbreviations: OR, Odds Ratio.

**Figure 3:**
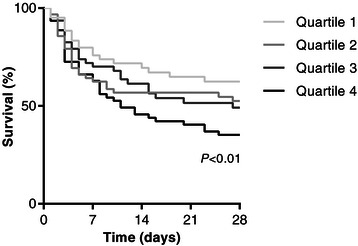
**28 day survival curves.** Patients with higher NT-proBNP levels died earlier and more frequently than patients with lower levels (log rank test for trend, *P* < 0.01).

Based on the NT-proBNP levels measured upon admission, patients were grouped into quartiles. The first quartile consisted of patients with NT-proBNP levels below 203 ng/l, the second quartile of patients with values between 203 and 787 ng/l, the third quartile of patients with values between 787 and 2807 ng/l and the fourth quartile was populated by patients with levels higher than 2807 ng/l. Using a univariate binary logistic regression model, NT-proBNP levels of the 4^th^ quartile were associated with increased odds of death within 28 days post ICU admission (OR 3.6, 95% CI = 1.7 – 7.5) (Table 3, Figure 2A). Also, survival rates were lower in the increasing quartiles (log rank test for trend, *P* < 0.01; Figure 3).

**Table 3:** **Binary logistic regression models for increasing NT-proBNP quartiles and mortality**

	Unadjusted				Adjusted			
	B^a^	S.E.^b^	*P* ^c^	OR^d^	B^a^	S.E.^b^	*P* ^c^	OR^d^
Quartile 1 (<203 ng/l)				1				1
Quartile 2 (203–787 ng/l)	0.519	0.372	0.163	1.7	0.090	0.417	0.829	1.1
Quartile 3 (787–2807 ng/l)	0.454	0.372	0.222	1.6	0.124	0.413	0.764	1.1
Quartile 4 (>2807 ng/l)	1.270	0.378	0.001	3.6	0.475	0.440	0.280	1.6
Age (years)					0.026	0.409	0.021	1.0
Diabetes Mellitus (1 = yes)					0.548	0.340	0.181	1.7
VF/VT as initial rhytm (1 = yes)					−0.723	0.392	0.033	0.5
Cardiac Cause of Arrest (1 = yes)					−0.485	0.720	0.215	0.6

^a^Regression coefficient.

^b^Standard Error.

^c^Two-tailed test.

^d^Odds ratio.

Adding age (*P* < 0.001), diabetes (*P =* 0.070), VF/VT (*P <* 0.001) and cardiac cause of arrest (*P* < 0.01) to the binary logistic regression as covariates resulted in loss of the association between the upper NT-proBNP quartiles and 28-day mortality (Table 3, Figure 2B). Witnessed arrest was not considered as a covariate due to its unknown effect on the resuscitation period. Also, APACHE II was disregarded because it is not readily available immediately upon ICU admission, but 24 hours later.

### Receiver operating characteristic curve

**Figure 4:**
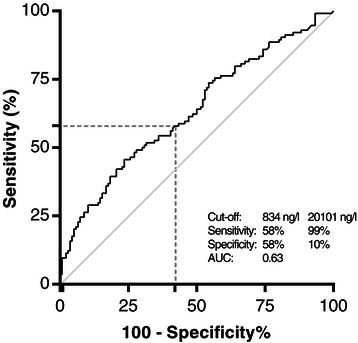
**NT-proBNP ROC curve.** An optimal sensitivity and specificity of respectively 58% and 58% was reached with a baseline NT-proBNP cut-off value of 834 ng/l. Corresponding positive and negative predictive values were 54% and 62%, respectively. When considering maximal specificity, a cut-off of 20101 ng/l yielded a specificity of 99% and a sensitivity of 10%. PPV and NPV were 92% and 57%, respectively. AUC 0.63 (95%CI 0.56 - 0.70; *P* < 0.0001).

The ROC curve (Figure 4) for baseline NT-proBNP showed that the highest combined sensitivity and specificity to predict 28-day mortality, was achieved using a cut-off value of 834 ng/l. This generated a sensitivity of 58% and a specificity of 58%. The area under the curve (AUC) was 0.63. The positive predictive value (PPV) was 54% (chance of death within 28 days with a baseline NT-proBNP value higher than 834 ng/l). The negative predictive value (NPV) was 62% (chance of survival with a baseline NT-proBNP value lower than 834 ng/l). The highest specificity of 99% was reached at a cut-off level of 20101 ng/l, which was accompanied by a sensitivity of 10%. The corresponding PPV and NPV were 92% and 57%, respectively. Using NT-proBNP values obtained during or after MTH did not improve prognostic performance (data not shown).

### Dynamics of NT-proBNP levels during mild therapeutic hypothermia

From the 250 patients, 139 patients had sequential NT-proBNP levels measured between the start of induction and the end of the rewarming phase (Figure 1). Patients who died during the induction, maintenance or rewarming phase (n = 26) were excluded to prevent possible severity of illness-based bias in consecutive comparisons, leaving 113 patients with NT-proBNP levels measured at all time points. Two specific expression patterns were observed over time; NT-proBNP levels either increased or decreased. Based on the pattern present, patients were grouped as being a Descender (n = 51) or an Ascender (n = 62) (Table 4).

**Table 4:** **MTH phases**

	Descender		Ascender	
	n = 51 (45%)		n = 62 (55%)	
Died (n)	21	(41%)	23	(37%)
**NT-proBNP**				
NT-proBNP, start induction (ng/l)	1981	(483–4217)	303	(116–1331)*†
NT-proBNP, start maintenance (ng/l)	2011	(720–4239)†	588	(213–1710)*†
NT-proBNP, end maintenance (ng/l)	1297	(454–3761)†	1591	(476–3448)
NT-proBNP, end rewarming (ng/l)	747	(287–2037)	1681	(472–3383)
**Induction phase**				
paO_2_ (mmHg)	96	(75–129)	88	(72–111)
sO_2_ (%)	97	(93–99)	97	(93–99)
Lactate (μgl/L)	2.0	(1.2–2.6)	2.5	(1.4–4.4)
Leukocyte count (10^9/L)	14.0	(11.3–18.5)	17.7	(12.3–21.8)
Heart Rate (bpm)	68	(58–76)	73	(59–91)
Systolic blood pressure (mmHg)	114	(104–129)	106	(100–117)*
Diastolic blood pressure (mmHg)	60	(57–68)	62	(54–69)
Noradrenaline (mg)	0.44	(0.02–1.15)	0.39	(0.08–1.11)
Diuresis (ml/hour)	74	(34–144)	67	(35–150)
**Maintenance phase**				
paO_2_ (mmHg)	86	(73–112)	88	(73–107)
sO_2_ (%)	97	(94–99)	96	(94–98)
Lactate (μgl/L)	1.4	(1.0–2.2)	2.1	(1.4–3.4)*
Leukocyte count (10^9/L)	10.4	(7.6–13.6)	13.0	(9.8–16.6)*
Heart Rate (bpm)	57	(48–66)	61	(50–71)
Systolic blood pressure (mmHg)	109	(104–117)	105	(101–111)
Diastolic blood pressure (mmHg)	57	(53–60)	57	(53–62)
Noradrenaline (mg)	0.61	(0.23–1.20)	0.60	(0.15–1.19)
Diuresis (ml/hour)	77	(44–123)	72	(34–116)
**Rewarming phase**				
paO_2_ (mmHg)	85	(74–100)	88	(76–102)
sO_2_ (%)	97	(95–98)	97	(93–98)
Lactate (μgl/L)	1.3	(0.9–2.1)	2.0	(1.5–2.8)*
Leukocyte count (10^9/L)	10.4	(7.5–15.2)	11.1	(9.2–16.3)
Heart Rate (bpm)	72	(63–85)	75	(66–86)
Systolic blood pressure (mmHg)	108	(100–115)	109	(100–119)
Diastolic blood pressure (mmHg)	55	(52–59)	55	(51–59)
Noradrenaline (mg)	0.81	(0.11–1.29)	0.61	(0.17–1.60)
Diuresis (ml/hour)	42	(30–68)	38	(21–86)

NT-proBNP, start induction; values measured between ICU admission and the start of cooling. NT-proBNP, start maintenance; the first value measured after reaching target temperature (32°C). NT-proBNP, end maintenance; the last value measured before initiation of the warming phase. NT-proBNP end-rewarming; the first value measured after reaching target temperature (36,5°C). * = *P* < 0.05 for inter-group comparisons (Mann Whitney U-test). † = *P* < 0.05 for intra-group comparison to the consecutive phase. MTH, mild therapeutic hypothermia; NT-proBNP, N-terminal pro Brain Natriuretic Peptide; ICU, Intensive Care Unit.

At the start of induction, median NT-proBNP levels of Descenders were 1981 ng/l (IQR 483–4217) and maintained at that level during the induction phase. At the end of the maintenance phase NT-proBNP levels significantly decreased compared to start of the maintenance phase and reached even significantly lower levels at the end of the rewarming phase. In the Ascenders group NT-proBNP levels increased significantly during the induction phase and further increased towards the end of the maintenance phase. During the rewarming phase NT-proBNP levels did not change compared to the end of the maintenance phase.

In comparison, Ascenders started with significantly lower NT-proBNP levels than Descenders at the start of the induction. This difference persisted until the end of the induction phase and was lost after the maintenance phase. Other than NT-proBNP, only the average systolic blood pressure during the induction phase was different between groups (Table 4). Ascenders had a significantly lower systolic blood pressure than Descenders*.* During the maintenance phase however, both lactate levels and leukocyte counts were higher in the Ascender group, compared to the Descender group. Lactate levels remained higher during the rewarming phase in the Ascender group compared to the Descender group. Other parameters, such as PaO_2_, sO_2_, heart rate, diastolic blood pressure and noradrenaline use was comparable between both groups during all phases of MTH. Additionally, the number of deaths in the Ascender and the Descender group did not differ significantly.

## Discussion

The present study shows that NT-proBNP levels upon ICU admission in cardiopulmonary resuscitated patients are positively associated with death within 28 days following the initial insult. Irrespective of other prognostic data, patients scaled into the 4^th^ NT-proBNP quartile had higher odds of dying, compared to patients in the lowest quartile. When adjusted for co-morbidities and arrest conditions however, statistical significance was lost. As an independent prognostic marker NT-proBNP achieved a mediocre optimal sensitivity and specificity of 58% with a cut-off value of 834 ng/l. With our study population, this translates into a negative predictive value of 62% and a positive predictive value of 54%. Alternatively, when one seeks to optimize specificity (cut-off at 20101 ng/l), NT-proBNP produced a PPV of 92% with a persistent low NPV at 57%. Due to the high spread in NT-proBNP levels, we think that measuring NT-proBNP at the ICU, utilizing either cut-off, is of little value for the prediction of outcome.

To our knowledge, this is the first study to evaluate the prognostic value of NT-proBNP in post-CA patients, treated with MTH. However, a study by Nagao *et al.* assessed the resuscitative value of BNP in post-CA patients treated with MTH [5]. Similarly, they established a positive relationship between higher BNP levels on admission at the emergency room and unfavourable outcome. Using a BNP cut off value of 80 pg/ml, they were able to predict an unfavourable neurological outcome with a sensitivity of 84% and a specificity of 89%. Their secondary endpoint survival to hospital discharge decreased in stepwise fashion across the increasing quartiles of BNP level. These results were later on confirmed by Sodeck *et al.,* showing BNP levels on admission to the emergency room predict neurological outcome and also survival at 6 months after CA [4]. As compared to our current investigation however, BNP performed better as an independent prognostic biomarker, as indicated by a higher sensitivity and specificity and a persisting significant odds ratio after multivariate regression analysis.

The above findings counter our hypothesis that NT-proBNP is of superior prognostic value in comparison to BNP. Several important differences should be noted. Fore one, we assessed 28-day mortality as a primary endpoint, while the other two studies documented neurological outcome and survival to hospital discharge or in a period of six months. More importantly, we measured NT-proBNP levels upon admission to the ICU, while BNP levels were determined upon arrival at the emergency department. The latter is possibly of special importance, because patients most likely have received therapeutic interventions that can influence NT-proBNP levels. One such intervention at our hospital is the infusion of cold saline at the emergency department as a preparation to MTH at the ICU.

Therapeutic intervention in itself, such as intravenous administration of fluids, oxygenation and vasopressors can influence BNP gene expression. The infusion of saline increases BNP transcription, without affecting blood pressure or heart rate [17]. Although the authors did not provide an explanation for this phenomenon, it is likely that infusion leads to increased ventricular expansion and subsequent synthesis of BNP. Additionally, infusion of fluids may lead to hemodilution, causing relative hypoxia. This is the opposite of one of the suggested biological functions of BNP, which is to facilitate hemoconcentration in order to increase the per volume oxygen carrying capacity of blood in response to hypoxia [18]. In contrast, the opposite of hypoxia, a higher than normal partial oxygen pressure, hyperoxia, may also cause elevation of NT-proBNP levels in-vivo [19]. Supplemental oxygen is often administered during cardiopulmonary resuscitation and a high PaO_2_ is no rare phenomenon, especially in mechanically ventilated patients [20-22]. In regard to vasopressor therapy, in-vitro experiments show that human cardiomyocytes synthesize BNP upon stimulation with either dobutamine, epinephrine or norepinephrine [23]. The BNP measurements in the study of Nagao *et al.* were all performed before administration of epinephrine [5].

Even though we cannot confirm that the abovementioned interventions actually cause a cumulative increase of NT-proBNP in post-CA patients, thereby possibly reducing prognostic performance, it warrants attention towards the application of NT-proBNP as a prognostic marker in an ICU setting. For future studies, it would therefore be interesting to acquire blood samples for NT-proBNP testing as early as possible, right after return of spontaneous circulation or at arrival at the emergency department and re-evaluate its prognostic value.

### Dynamics of NT-proBNP levels during mild therapeutic hypothermia

To the best of our knowledge this is the first study with repeated measurement of NT-proBNP plasma levels in post-CA patients. During MTH treatment, two types of responses where observed. NT-proBNP levels increased (Ascenders) or decreased (Descenders) over the course of MTH.

Descenders were admitted to the ICU with a significantly higher NT-proBNP value than Ascenders. In the case of NT-proBNP decrease in time, we saw that levels continue to drop even after rewarming. In the Ascender group however, the rise of NT-proBNP had stopped at the end of the maintenance phase. Interestingly, the differences between Ascenders and Descenders, in regard to NT-proBNP levels, were completely lost at the end of the maintenance phase. This may indicate that the application of hypothermia imposes a similar state in all patients. Which for some patients means relieve (NT-proBNP levels decrease), while in others MTH may actually cause stress (NT-proBNP levels increase). In accordance, MTH is currently applied as a one-size fits all therapy, with a target temperature of 32.5°C for all patients. The increase of NT-proBNP levels in the Ascender group could also be due to an insufficient control of inflammation, relative to the Descender group. Although there was insufficient inflammation related data available to properly investigate this relationship, leukocyte counts did appear to be higher overall in the Ascender group. Ultimately, the different responses did not appear to affect outcome. In other words, an increase or decrease of NT-proBNP levels was not associated with 28-day mortality. Overall, we see the response-heterogeneity in patients treated with MTH as a confirmation that therapy induced changes in NT-proBNP levels do not necessarily reflect severity of illness, indicated by a lack of association with 28-day mortality, and thereby reduce overall sensitivity and specificity of NT-proBNP as predictive marker.

### Study limitations

The small study population is one of the limitations of the current study. Implementation of age adjusted cut-off values might improve the predictive performance of NT-proBNP, but this requires a considerably larger sample size. Mortality as an endpoint might also be considered inadequate, since survival does not guarantee a subsequent satisfactory quality of life. A quality of life questionnaire or inclusion of a cerebral performance category score on hospital discharge in a prospective follow-up study would address this issue.

This was a retrospective study and not all patients had sequential NT-proBNP levels measured. As often with retrospective studies, it is difficult to determine why these measurements were omitted in these particular patients and as a result may have caused selection bias in our analysis.

Additionally, the data used in this analysis were gathered prior to the publication by Nielsen et al., who compared different target temperatures [24]. Our ICU only applies a target temperature of 32.5°C and therefore the dynamics of NT-proBNP during MTH reported here cannot be extrapolated to treatment with a higher target temperature without confirmation in an additional study.

Since measurements were done at the ICU, therapeutic interventions at the emergency department, such as fluid infusions and vasopressor therapy, may have siginificantly influenced the prognosticating ability of NT-proBNP.

## Conclusions

High NT-proBNP plasma concentrations on ICU admission are associated with high 28-day mortality in post-CA patients treated with MTH in a univariate analysis. However, when considering age, presence of diabetes, VF/VT and cause of cardiac arrest in a multivariate analysis, NT-proBNP offers no additional prognostic information. During MTH, an overall increase or decrease of NT-proBNP levels was observed, which appear to be unrelated to 28-day mortality.

## Abbreviations

BNP: B-type natriuretic peptide
CA: Cardiac arrest
CAG: Coronary Angiogram
CHF: Congestive heart failure
CK: Creatine Kinase
CVD: Cardiovascular disease
DM: Diabetes mellitus
ICU: Intensive Care Unit
MB: Myoglobin
MTH: Mild therapeutic hypothermia
NPPB: Natriuretic peptide precursor B
NT-proBNP: N-terminal pro Natriuretic Peptide
ROC: Receiver operating characteristic
VF: Ventricular fibrillation
VT: Ventricular tachycardia

## References

[1] Peberdy MA, Callaway CW, Neumar RW, Geocadin RG, Zimmerman JL, Donnino M (2010). Part 9: post-cardiac arrest care: 2010 American Heart Association Guidelines for Cardiopulmonary Resuscitation and Emergency Cardiovascular Care. Circulation..

[2] Berdowski J, Berg RA, Tijssen JG, Koster RW (2010). Global incidences of out-of-hospital cardiac arrest and survival rates: Systematic review of 67 prospective studies. Resuscitation..

[3] Scolletta S, Donadello K, Santonocito C, Franchi F, Taccone FS (2012). Biomarkers as predictors of outcome after cardiac arrest. Expert Rev Clin Pharmacol..

[4] Sodeck GH, Domanovits H, Sterz F, Schillinger M, Losert H, Havel C (2007). Can brain natriuretic peptide predict outcome after cardiac arrest? An observational study. Resuscitation..

[5] Nagao K, Mukoyama T, Kikushima K, Watanabe K, Tachibana E, Iida K (2007). Resuscitative value of B-type natriuretic peptide in comatose survivors treated with hypothermia after out-of-hospital cardiac arrest due to cardiac causes. Circ J..

[6] Levin ER, Gardner DG, Samson WK (1998). Natriuretic peptides. N Engl J Med..

[7] Casals G, Ros J, Sionis A, Davidson MM, Morales-Ruiz M, Jimenez W (2009). Hypoxia induces B-type natriuretic peptide release in cell lines derived from human cardiomyocytes. Am J Physiol Heart Circ Physiol..

[8] Weidemann A, Klanke B, Wagner M, Volk T, Willam C, Wiesener MS (2008). Hypoxia, via stabilization of the hypoxia-inducible factor HIF-1alpha, is a direct and sufficient stimulus for brain-type natriuretic peptide induction. Biochem J..

[9] Due-Andersen R, Pedersen-Bjergaard U, Hoi-Hansen T, Olsen NV, Kistorp C, Faber J (2008). NT-pro-BNP during hypoglycemia and hypoxemia in normal subjects: impact of renin-angiotensin system activity. J Appl Physiol..

[10] LaPointe MC (2005). Molecular regulation of the brain natriuretic peptide gene. Peptides..

[11] Ogawa T, de Bold AJ (2012). Brain natriuretic Peptide production and secretion in inflammation. J Transplant..

[12] Anwaruddin S, Lloyd-Jones DM, Baggish A, Chen A, Krauser D, Tung R (2006). Renal function, congestive heart failure, and amino-terminal pro-brain natriuretic peptide measurement: results from the ProBNP Investigation of Dyspnea in the Emergency Department (PRIDE) Study. J Am Coll Cardiol..

[13] Giannakoulas G, Hatzitolios A, Karvounis H, Koliakos G, Charitandi A, Dimitroulas T (2005). N-terminal pro-brain natriuretic peptide levels are elevated in patients with acute ischemic stroke. Angiology..

[14] Yip HK, Sun CK, Chang LT, Chen MC, Liou CW (2006). Time course and prognostic value of plasma levels of N-terminal pro-brain natriuretic peptide in patients after ischemic stroke. Circ J..

[15] Etgen T, Baum H, Sander K, Sander D (2005). Cardiac troponins and N-terminal pro-brain natriuretic peptide in acute ischemic stroke do not relate to clinical prognosis. Stroke..

[16] Ordonez-Llanos J, Collinson PO, Christenson RH (2008). Amino-terminal pro-B-type natriuretic peptide: analytic considerations. Am J Cardiol..

[17] Heringlake M, Heide C, Bahlmann L, Eichler W, Pagel H, Schmucker P (2004). Effects of tilting and volume loading on plasma levels and urinary excretion of relaxin, NT-pro-ANP, and NT-pro-BNP in male volunteers. J Appl Physiol..

[18] Arjamaa O, Nikinmaa M (2011). Hypoxia regulates the natriuretic peptide system. Int J Physiol Pathophysiol Pharmacol..

[19] Yildiz S, Uzun G, Uz O, Ipcioglu OM, Kardesoglu E, Ozcan O (2008). N-terminal pro-B-type natriuretic peptide levels increases after hyperbaric oxygen therapy in diabetic patients. Clin Invest Med..

[20] de Graaff AE, Dongelmans DA, Binnekade JM, de Jonge E (2011). Clinicians’ response to hyperoxia in ventilated patients in a Dutch ICU depends on the level of FiO2. Intensive Care Med..

[21] Cornet AD, Kooter AJ, Peters MJ, Smulders YM (2012). Supplemental oxygen therapy in medical emergencies: more harm than benefit?. Arch Intern Med..

[22] Spindelboeck W, Schindler O, Moser A, Hausler F, Wallner S, Strasser C (2013). Increasing arterial oxygen partial pressure during cardiopulmonary resuscitation is associated with improved rates of hospital admission. Resuscitation..

[23] Valette X, Lemoine S, Allouche S, Gerard JL, Hanouz JL (2012). Effect of lipopolysaccharide, cytokines, and catecholamines on brain natriuretic peptide release from human myocardium. Acta Anaesthesiol Scand..

[24] Nielsen N, Wetterslev J, Cronberg T, Erlinge D, Gasche Y, Hassager C (2013). Targeted temperature management at 33 degrees C versus 36 degrees C after cardiac arrest. N Engl J Med..

